# Be Up and Doing

**Published:** 1901-01

**Authors:** 


### EDITORIAL.

### BE UP AND DOING.

In many States the law-making bodies are now in session, and it is important that veterinarians shall be fully alive to their duty in watching every step of legislation that is to measure their sphere of usefulness in every community as well as to restrict or advance the progress of veterinary science along the lines of sanitary medicine. Hardly a legislative body is now in session that has not before it some measures that involve the interests of veterinarians; in food-inspection laws, health measures, veterinary sanitary control work, livestock industrial interests, veterinary laws for regulating practice, the interests of veterinary colleges, laws to regulate educational work among colleges and universities—all of which bear deeply upon the interests of the veterinarians, and they should be alert in their support or opposition to these measures as they tend to advance or degrade true progress. Every county Secretary should procure from their representatives in legislative halls the legislative records, and should scan them carefully, and these officials should make up a legislative committee in every State and thus be in position to work unitedly in these matters.

